# Development and validation of prognostic models for small cell lung cancer patients with liver metastasis: a SEER population-based study

**DOI:** 10.1186/s12890-023-02832-7

**Published:** 2024-01-04

**Authors:** Qiaofeng Li, Yandong Zhao, Zheng Xu, Yongqing Ma, Chengyu Wu, Huayue Shi

**Affiliations:** 1 College of Traditional Chinese Medicine & College of Integrated Chinese and Western Medicine, Nanjing University of Chinese Medicine, No. 138 Xianlin Avenue, Qixia District, Nanjing, 210023 P. R. China; 2 Department of Science and Technology, The Affiliated Hospital of Nanjing University of Chinese Medicine, Nanjing, 210029 P. R. China

**Keywords:** Prognostic models, Small cell lung cancer patients, Liver metastasis, Nomogram, SEER database

## Abstract

**Background:**

This study was to establish and validate prediction models to predict the cancer-specific survival (CSS) and overall survival (OS) of small-cell lung cancer (SCLC) patients with liver metastasis.

**Methods:**

In the retrospective cohort study, SCLC patients with liver metastasis between 2010 and 2015 were retrospectively retrieved from the Surveillance, Epidemiology, and End Results (SEER) database. Patients were randomly divided into the training group and testing group (3: 1 ratio). The Cox proportional hazards model was used to determine the predictive factors for CSS and OS in SCLC with liver metastasis. The prediction models were conducted based on the predictive factors. The performances of the prediction models were evaluated by concordance indexes (C-index), and calibration plots. The clinical value of the models was evaluated by decision curve analysis (DCA).

**Results:**

In total, 8,587 patients were included, with 154 patients experiencing CSS and 154 patients experiencing OS. The median follow-up was 3 months. Age, gender, marital status, N stage, lung metastases, multiple metastases surgery of metastatic site, chemotherapy, and radiotherapy were independent predictive factors for the CSS and OS of SCLC patients with liver metastasis. The prediction models presented good performances of CSS and OS among patients with liver metastasis, with the C-index for CSS being 0.724, whereas the C-index for OS was 0.732, in the training set. The calibration curve showed a high degree of consistency between the actual and predicted CSS and OS. DCA suggested that the prediction models provided greater net clinical benefit to these patients.

**Conclusion:**

Our prediction models showed good predictive performance for the CSS and OS among SCLC patients with liver metastasis. Our developed nomograms may help clinicians predict CSS and OS in SCLC patients with liver metastasis.

**Supplementary Information:**

The online version contains supplementary material available at 10.1186/s12890-023-02832-7.

## Background

Lung cancer is one of the most common malignancies, and its morbidity and mortality are increasing worldwide [1, 2]. Small-cell lung cancer (SCLC) is a type of aggressive malignancy with rapid growth and early metastasis, accounting for between 10 and 15% of all lung cancer diagnoses [3]. Approximately two-thirds of SCLC patients have obvious metastasis at the time of clinical diagnosis [4]. Liver metastasis is one of the frequent metastatic sites of SCLC [5, 6]. SCLC patients with liver metastasis tend to have a worse prognosis, with the one-year survival rate being less than 20% [5, 7]. Identifying the prognostic factors of SCLC patients with liver metastasis and improving the prognosis of patients is currently needed.

Few studies have investigated the prognostic factors of SCLC with metastasis, although several studies have focused on the survival outcomes of patients with SCLC [8, 9]. According to a study, age was revealed to be a significant predictor of overall survival (OS) in SCLC patients with distant metastases [10]. Based on the study by Cheng et al., targeted therapy may play a significant role in improving patient prognosis in SCLC with liver metastases [11]. The factors associated with OS and cancer-specific survival (CSS) of the SCLC with liver metastasis warrant further investigation. The clinical prediction model is a significantly important tool that can stratify patients before treatment to determine whether a specific treatment scheme is worthy of implementation, which has been widely used in clinical practice [12]. Recently several prediction models have been developed to predict the survival in SCLC [9, 13, 14]. Nevertheless, most of the previous prediction models focused on early-stage, III-stage, and N2-stage SCLC [13, 15]. There is a scanty study focusing on the prognosis of liver metastasis in patients with SCLC [12]. Given the high mortality rate following liver metastases from SCLC and the various clinical characteristics of different patients with SCLC, prediction models for the prognosis in SCLC patients with liver metastases are imperative.

Herein, we investigated the factors associated with survival of SCLC with liver metastasis using a large cohort from the Surveillance, Epidemiology, and End Results (SEER) database and developed prediction models to predict their CSS and OS. Besides, we also verified the prediction models using internal validation and performed a series of tests to evaluate the performances of predictive models. The development of prediction models may enable a better treatment stratification for SCLC patients with liver metastasis.

## Methods

### Study design and participants

The retrospective cohort study was based on the SEER program which covers approximately 30% of the total US population [16]. The records of SCLC patients with liver metastasis between 2010 and 2015 were extracted from the database ‘SEER 18 Regs Custom Data (including additional treatment fields), November 2018 sub (1975–2016 varying) database using SEER*stat 8.3.5 software. The International Classification of Diseases for Oncology third edition (ICD-O-3) was used to identify SCLC by site codes [8002, 8041, 8043, 8144, 8145] [17]. The inclusion criteria were as follows: (I) SCLC was the only primary cancer; (II) the staging of lymph nodes followed the 7th edition of the American Joint Committee on 2 Journal of International Medical Research Cancer; (III) aged ≥ 18 years old; (IV) patients with liver metastasis [SEER Combined Mets at DX-liver (2010 +)]. Since the clinical data in this study were collected from a publicly available database, there were no local or state ethical issues. In addition, because this retrospective study was based on public data from the SEER database, informed consent was not required. All methods were performed in accordance with the relevant guidelines and regulations.

### Study variables and outcomes

The database reviewed retrospectively consisted of clinical characteristics of patients, pathological characteristics of tumors, and survival time (months). Continuous variables were transformed into categorical variables based on recognized cut-off values (for age). Clinical characteristics of patients included gender (female vs male), age (≤ 65 years, > 65 years), race (black, white, and other), and marital status (married and other). Pathological characteristics of tumors include primary site [multiple sites, upper lobe, middle lobe, lower lobe, main bronchus, not otherwise specified (NOS)], tumor size (≤ 24 mm, 25–37 mm, 38–59 mm, ≥ 60 mm), T stage in 7th edition AJCC system [T1, T2, T3, T4, and not specific known T stage (Tx)], N stage in 7th edition AJCC system [N1, N2, N3, N4, and not specific known N stage (Nx)], metastatic sites (bone, brain, lung, multiple other metastases, no metastases or unknown), surgery of primary site or not/unknown, surgery of metastatic site or not/unknown, radiotherapy or not/unknown, chemotherapy or not/unknown. The outcomes in this study were 1- and 2-year CSS and 1- and 2-year OS. The classification of tumor size was based on the inter-quartiles ranges. The outcomes in this study were 1- and 2-year CSS and 1- and 2-year OS. CSS was defined as the time between diagnosis and death owing to specific cancer during follow-up and OS was defined as death regardless of any causes. The total observation period was 2 years; follow-up would terminate if the patient died. The median follow-up time was 3 months.

### Development and validation of the prediction models

For the development of the nomograms, all of the 8,587 cases were randomly divided into the training set and test set (ratio: 3:1) by using the random-number generation method. The prediction models were established on the basis of the predictive factors identified by Cox proportional hazards model. The performances of the prediction models were evaluated by measuring the concordance index (C-index),calibration plots, and decision curve analysis (DCA).

### Statistical analysis

Measurement data by normal distribution are described in mean ± standard deviation (Mean ± SD). The independent sample t-test or analysis of one-way variance (ANOVA) was used for comparisons between groups. Non-normal data were described as a median and interquartile range [M (Q_1_, Q_3_)], and the Mann–Whitney U test or Kruskal–Wallis test was applied for comparisons between groups. Enumeration data were described as the number of cases and the constituent ratio [N (%)]. The Chi-square test was used for comparison between groups.

Hazard ratios (HRs) and their 95% confidence intervals (CIs) for each potential predictive variable were analyzed using Cox proportional risk models. The variables with multiple categories were transformed into dummy variables before further analyses. False-discovery rate (FDR) adjusted *P* values were calculated to correct for multiple testing. Based on the predictive factors, prediction models were conducted to predict the 1- and 2-year CSS and OS. The performances of the final nomogram were assessed by C-index, and calibration measures as the measuring tool. The C-index is a concordance measure analogous to ROC, which values range from 0.5 (no discrimination) to 1.0 (perfect discrimination). The higher the value between 0.5 and 1, the stronger the resolution of the nomogram. Calibration measures to what degree the predicted probabilities are close to actual outcomes. Calibration plots of the nomogram for 1- and 2-year CSS and OS were performed in the training set and the testing set. The prediction models were also verified by 5-fold cross validation. The 5-fold cross-validation was done to make the results more realistic and to avoid chance. The data set was divided into five groups by a 5-fold cross-validation operation. For each training session, one set was used as the validation set and the remaining four sets were used as the training set. After addressing the ability of the nomogram, we used DCA to test the reliability of the model, which was a method for evaluating alternative diagnostic and prognostic strategies that have advantages over other commonly used measures and techniques. If the threshold probability of net benefits is unpractical, then the applicability of a well-performing model may be limited, meaning that the benefits of the new prediction model will be less than the benefits of existing tools, and may even be detrimental. The prediction models with and without chemotherapy for predicting CSS and OS in SCLC patients with liver metastasis were conducted and were compared with the prediction models. Data analysis used R software version 4.1.2 (R Foundation). All tests were two-tailed and *P* < 0.05 was considered statistically significant,

## Results

### Characteristics of the included patients

**Fig. 1:**
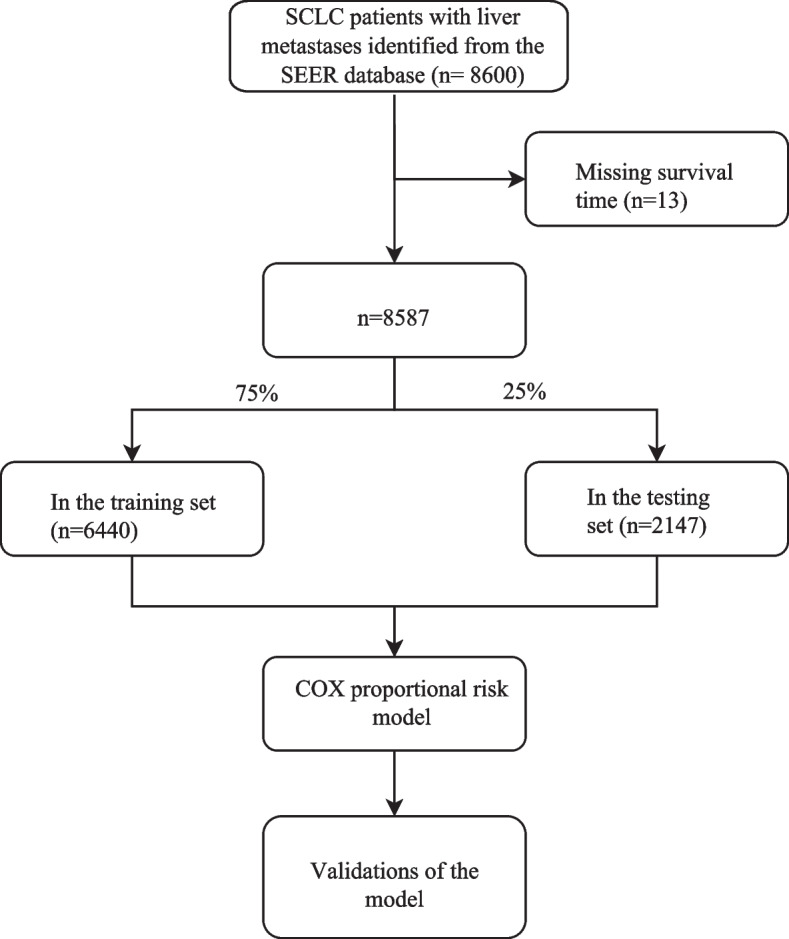
The flow chart of patient’s selection

In total, 8,587 eligible patients, who were diagnosed as SCLC patients with liver metastasis from 2010 to 2015 were identified in the SEER database. The flow chart of patient’s selection is shown in Fig. 1. Among them, 154 patients experienced CSS, and 154 patients experienced OS. The median follow-up time was 3 months. For all patients, there were 3,770 (43.90%) patients with age ≤ 65 years and 4,817 (56.10%) patients with age > 65 years. As for the race, the majority of patients (88.84%) were white. With respect to the primary site, most of them were located in the upper lobe. The majority of patients were classified as T4 (33.42%) and N2 (55.65%). The baseline characteristics of the patients are presented in Table 1.

**Table 1:** Demographic and clinical characteristics in SCLC patients with liver metastasis

Variables	Total (*n* = 8,587)	Training set (*n* = 6,440)	Testing set (*n* = 2,147)
Age, n (%), years			
≤ 65	3770 (43.90)	2796 (43.42)	974 (45.37)
> 65	4817 (56.10)	3644 (56.58)	1173 (54.63)
Gender, n (%)			
Female	3988 (46.44)	2947 (45.76)	1041 (48.49)
Male	4599 (53.56)	3493 (54.24)	1106 (51.51)
Race, n (%)			
Black	663 (7.72)	491 (7.62)	172 (8.01)
White	7629 (88.84)	5731 (88.99)	1898 (88.40)
Others	295 (3.44)	218 (3.39)	77 (3.59)
Marital status, n (%)			
Other	4300 (50.08)	3223 (50.05)	1077 (50.16)
Married	4287 (49.92)	3217 (49.95)	1070 (49.84)
Primary sites, n (%)			
Multiple	134 (1.56)	95 (1.48)	39 (1.82)
Upper lobe	3698 (43.07)	2797 (43.43)	901 (41.97)
Middle lobe	262 (3.05)	205 (3.18)	57 (2.65)
Lower lobe	1753 (20.41)	1309 (20.33)	444 (20.68)
Main bronchus	937 (10.91)	701 (10.89)	236 (10.99)
NOS	1803 (21.00)	1333 (20.70)	470 (21.89)
Tumor sizes, n (%)			
0–24 mm	2036 (23.71)	1527 (23.71)	509 (23.71)
25–37 mm	2193 (25.54)	1648 (25.59)	545 (25.38)
38–59 mm	2184 (25.43)	1634 (25.37)	550 (25.62)
≥ 60 mm	2174 (25.32)	1631 (25.32)	543 (25.29)
T stage, n (%)			
T0	105 (1.22)	77 (1.20)	28 (1.30)
T1	640 (7.45)	471 (7.31)	169 (7.87)
T2	1835 (21.37)	1393 (21.63)	442 (20.59)
T3	1603 (18.67)	1180 (18.32)	423 (19.70)
T4	2870 (33.42)	2165 (33.62)	705 (32.84)
Tx	1534 (17.86)	1154 (17.92)	380 (17.70)
N stage, n (%)			
N0	890 (10.36)	678 (10.53)	212 (9.87)
N1	519 (6.04)	5.82 (16.35)	144 (6.71)
N2	4779 (55.65)	3564 (55.34)	1215 (56.59)
N3	1849 (21.53)	1399 (21.72)	450 (20.96)
Nx	550 (6.41)	424 (6.58)	126 (5.87)
Metastatic lesion, n (%)			
Bone	2153 (25.07)	1599 (24.83)	554 (25.80)
Brain	565 (6.58)	419 (6.51)	146 (6.80)
Lung	729 (8.49)	537 (8.34)	192 (8.94)
Multiple metastases	1527 (17.78)	1147 (17.81)	380 (17.70)
No or unknown	3613 (42.08)	2738 (42.52)	875 (40.75)
Surgery of primary site, n (%)			
No/unknown	8553 (99.60)	6414 (99.60)	2139 (99.63)
Yes	34 (0.40)	26 (0.40)	8 (0.37)
Surgery of metastatic site, n (%)			
Not/unknown	8416 (98.01)	6309 (97.97)	2107 (98.14)
Yes	171 (1.99)	131 (2.03)	40 (1.86)
Chemotherapy, n (%)			
Not/unknown	3059 (35.62)	2292 (35.59)	767 (35.72)
Yes	5528 (64.38)	4148 (64.41)	1380 (64.28)
Radiotherapy, n (%)			
Not/unknown	6216 (72.39)	4664 (72.42)	1552 (72.29)
Yes	2371 (27.61)	1776 (27.58)	595 (27.71)
CSS	0.0179	0.0174	0.0196
OS	1.79%	1.74%	1.96%

*SCLC* small cell lung cancer, *NOS* not otherwise specified, *Tx* not specific known T stage, *Nx* not specific known N stage, *CSS* cancer specific survival, *OS* overall survival

### Identification of the predictive factors for CSS and OS in SCLC patients with liver metastasis

Identifications of factors associated with CSS and OS are shown in Table 2. The results demonstrated that age > 65 years (HR: 1.303, 95% CI: 1.238–1.372, *P* < 0.001), being male (HR: 1.113, 95% CI: 1.057–1.171, *P* < 0.001), being married (HR: 0.907, 95% CI: 0.863–0.955, *P* < 0.001), Nx stage (HR: 1.264, 95% CI: 1.115–1.433,* P* < 0.001, FDR-adjusted *P* < 0.001), lung metastases (HR: 1.189, 95% CI: 1.075–1.316, *P* = 0.001, FDR-adjusted *P* = 0.004), multiple metastases (HR: 1.115, 95% CI: 1.031–1.206, *P* = 0.007 FDR-adjusted *P* = 0.021), surgery of metastatic site (HR: 0.927, 95% CI: 0.776–1.109, *P* = 0.407), chemotherapy (HR: 0.232, 95% CI: 0.219–0.246, *P* < 0.001), and radiotherapy (HR: 0.627, 95% CI: 0.593–0.664, *P* < 0.001) were associated with CSS in SCLC patients with liver metastasis. Age > 65 years, being male, being married, Nx stage, lung metastases, multiple metastases, no metastases/unknown, surgery of metastatic site, chemotherapy, and radiotherapy were associated with OS in SCLC patients with liver metastasis.

**Table 2:** Identifications of factors associated with CSS and OS in SCLC patients with liver metastasis

Variables	CSS			OS		
	HR (95% CI)	*P*	FDR-adjusted *P*	HR (95% CI)	*P*	FDR-adjusted *P*
Age, n (%), years						
≤ 65	1			1		
> 65	1.303 (1.238–1.372)	< 0.001		1.325 (1.261–1.393)	< 0.001	
Gender, n (%)						
Female	1			1		
Male	1.113 (1.057–1.171)	< 0.001		1.135 (1.080–1.192)	< 0.001	
Race, n (%)						
Black	1			1		
White	1.068 (0.970–1.177)	0.178		1.050 (0.956–1.153)	0.305	
Others	0.971 (0.822–1.147)	0.733		0.973 (0.829–1.143)	0.741	
Marital status, n (%)						
Others	1			1		
Married	0.907 (0.863–0.955)	< 0.001		0.896 (0.853–0.942)	< 0.001	
Primary sites, n (%)						
Multiple sites	1			1		
Upper lobe	0.932 (0.756–1.148)	0.508		0.935 (0.762–1.147)	0.518	
Middle lobe	0.877 (0.683–1.126)	0.304		0.894 (0.701–1.141)	0.369	
Lower lobe	0.916 (0.740–1.134)	0.420		0.931 (0.756–1.146)	0.500	
Main bronchus	0.894 (0.718–1.113)	0.315		0.890 (0.718–1.103)	0.286	
NOS	1.087 (0.879–1.345)	0.441		1.101 (0.894–1.356)	0.366	
Tumor size, n (%)						
0–24 mm	1			1		
25–37 mm	1.025 (0.954–1.102)	0.498		1.030 (0.960–1.106)	0.404	
38–59 mm	1.018 (0.947–1.094)	0.634		1.021 (0.951–1.095)	0.572	
≥ 60 mm	0.944 (0.878–1.015)	0.120		0.948 (0.883–1.017)	0.135	
T, n (%)						
T0	1			1		
T1	0.925 (0.718–1.191)	0.544		0.906 (0.710–1.155)	0.424	
T2	1.018 (0.800–1.295)	0.885		0.981 (0.779–1.236)	0.873	
T3	1.068 (0.839–1.360)	0.594		1.034 (0.820–1.305)	0.775	
T4	1.004 (0.791–1.274)	0.976		0.972 (0.773–1.223)	0.811	
Tx	1.169 (0.918–1.489)	0.206		1.136 (0.900–1.433)	0.284	
N, n (%)						
N0	1			1		
N1	0.959 (0.841–1.093)	0.528	0.628	0.945 (0.832–1.074)	0.387	0.592
N2	0.979 (0.899–1.066)	0.628	0.628	0.978 (0.900–1.062)	0.592	0.592
N3	0.923 (0.839–1.015)	0.097	0.291	0.926 (0.844–1.017)	0.107	0.321
Nx	1.264 (1.115–1.433)	< 0.001	< 0.001	1.267 (1.121–1.431)	< 0.001	< 0.001
Metastatic sites, n (%)						
Bone	1			1		
Brain	1.116 (1.000–1.247)	0.051	0.051	1.110 (0.996–1.237)	0.059	0.059
Lung	1.189 (1.075–1.316)	0.001	0.004	1.206 (1.092–1.331)	< 0.001	< 0.001
Multiple metastases	1.115 (1.031–1.206)	0.007	0.021	1.118 (1.036–1.207)	0.004	0.012
No metastases/unknown	1.068 (1.002–1.139)	0.042	0.051 0	1.087 (1.021–1.157)	0.009	0.018
Surgery of primary site, n (%)						
Not/unknown	1			1		
Yes	0.652 (0.440–0.966)	0.033		0.619 (0.418–0.916)	0.017	
Surgery of metastatic site, n (%)						
Not/unknown	1			1		
Yes	0.927 (0.776–1.109)	0.407		0.938 (0.789–1.116)	0.471	
Chemotherapy, n (%)						
Not/unknown	1			1		
Yes	0.232 (0.219–0.246)	< 0.001		0.225 (0.213–0.238)	< 0.001	
Radiotherapy, n (%)						
Not/unknown	1			1		
Yes	0.627 (0.593–0.664)	< 0.001		0.616 (0.583–0.651)	< 0.001	

*SCLC* small cell lung cancer, *NOS* not otherwise specified, *CSS* cancer specific survival, *HR* hazard ratio, *CI* confidence interval, *FDR* false-discovery rate, *OS* overall survival, *Tx* not specific known T stage, *Nx* not specific known N stage

### Development of prediction models for CSS and OS in SCLC patients with liver metastasis

**Fig. 2:**
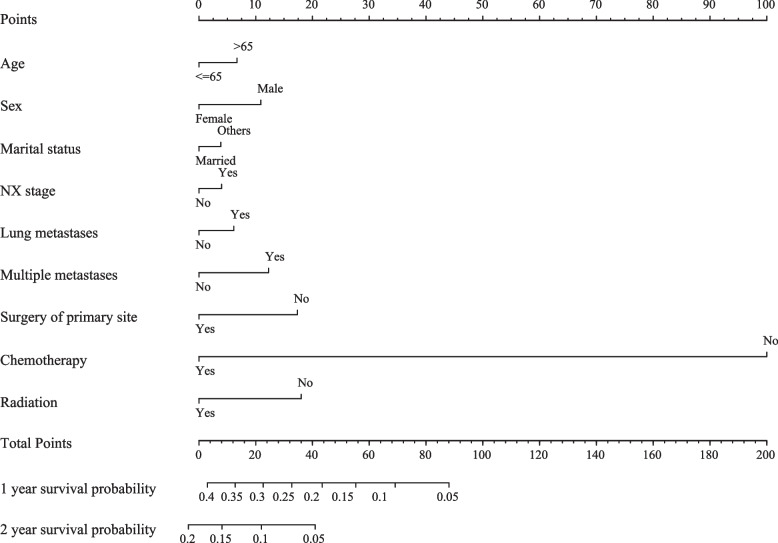
The nomogram containing independent predictive factors for the 1- and 2-year CSS and OS prediction of SCLC patients with liver metastasis; CSS: cancer-specific survival; SCLC: small-cell lung cancer

**Fig. 3:**
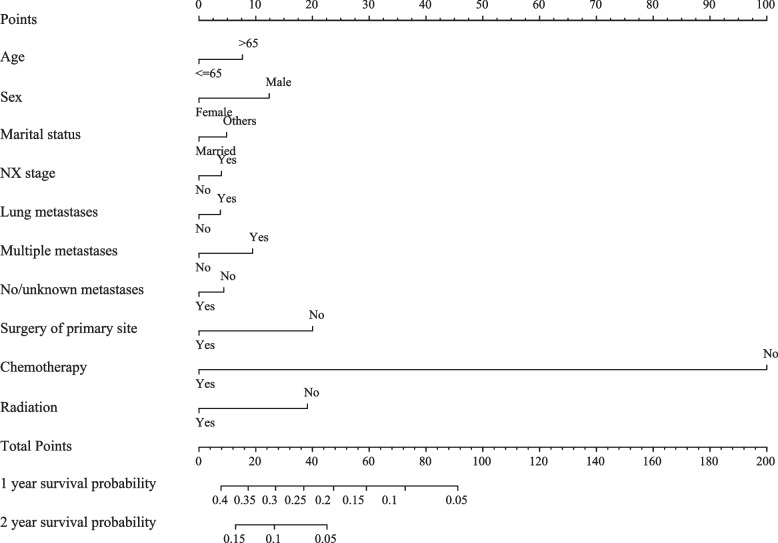
The nomogram containing independent predictive factors for the 1- and 2-year OS prediction of SCLC patients with liver metastasis; OS: overall survival; SCLC: small-cell lung cancer

We developed two nomograms respectively for CSS and OS. Each of the variables was given a point according to the HR. Then, by adding the total score of each variable and locating the score on the total points scale, the 1- and 2-year CSS and OS could be obtained. The nomograms containing independent predictive factors for predicting 1- and 2-year CSS and OS prediction of SCLC patients with liver metastasis are shown in Figs. 2 and 3.

### Performance of the prediction models for CSS and OS in SCLC patients with liver metastasis

**Fig. 4:**
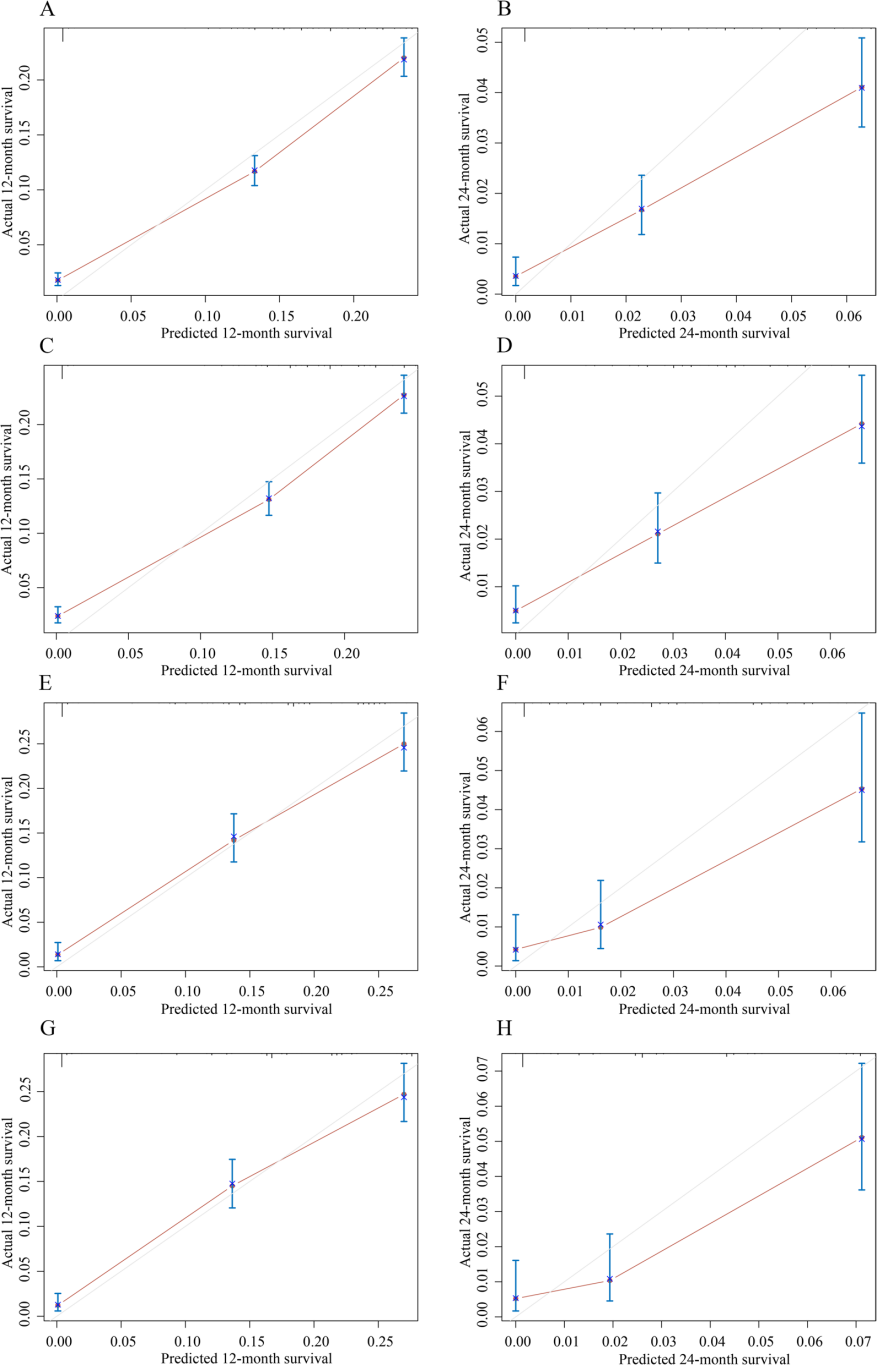
The calibration curve for the 1- and 2-year CSS and OS prediction of SCLC patients with liver metastasis; **A** 1-year CSS in the training set; **B** 2-year CSS in the training set; **C** 1-year OS in the training set; **D** 2-year OS in the training set; **E** 1-year CSS in the test set; **F** 2-year CSS in the test set; **G** 1-year OS in the test set; **H** 2-year OS in the test set. CSS: cancer-specific survival; OS: overall survival; SCLC: small-cell lung cancer

**Fig. 5:**
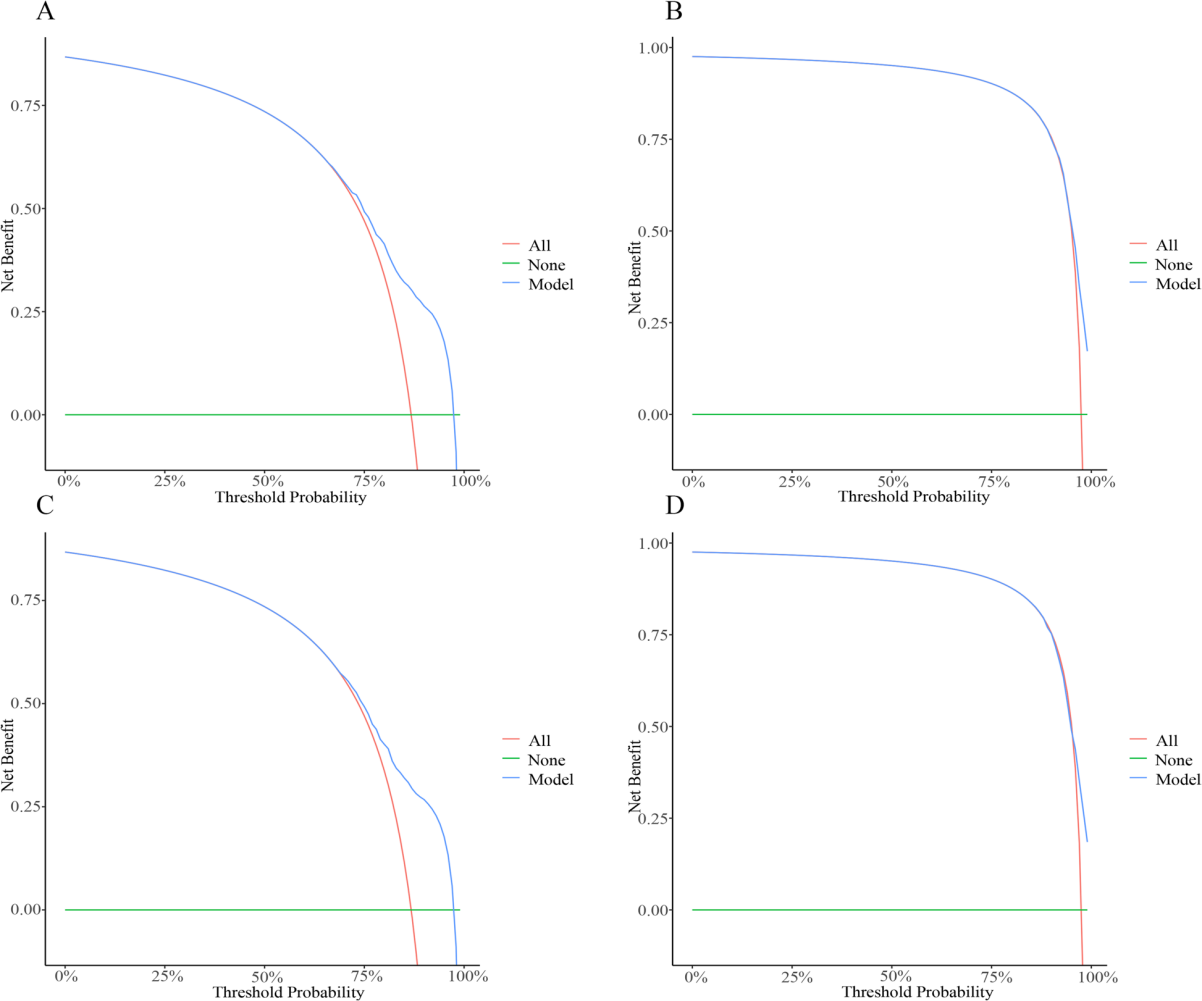
The DCA curve for the 1- and 2-year CSS and OS prediction of SCLC patients with liver metastasis; **A** 1-year CSS in the training set; **B** 2-year CSS in the training set; **C** 1-year OS in the training set; **D** 2-year OS in the training set. In the figure, the abscissa is the threshold probability, the ordinate is the net benefit rate. The horizontal green one indicates that all samples are negative and all are not treated, with a net benefit of zero. The oblique red one indicates that all samples are positive. The net benefit is a backslash with a negative slope (blue). DCA: decision curve analysis; CSS: cancer-specific survival; OS: overall survival; SCLC: small-cell lung cancer

In the training set, the C-index for CSS predicted by the prediction model was 0.724 (95% CI: 0.716–0.731), whereas the C-index for OS was 0.732 (95% CI: 0.724–0.739) (Table 3). In the testing set, the C-index for CSS and OS were 0.734 (95% CI: 0.722–0.747), and 0.739 (95% CI: 0.727–0.751), respectively. The C-index of the prediction model for predicting 1-year CSS, and 2-year CSS was 0.727 (95% CI: 0.719–0.734), and 0.724 (95% CI: 0.717–0.731), respectively, in the training set. The C-index of the prediction model for predicting 1-year OS, and 2-year OS was 0.734 (95% CI: 0.727–0.742), and 0.732 (95% CI: 0.725–0.739), respectively, in the training set. C-indexes of the prediction models for CSS and OS in SCLC patients with liver metastasis are presented in Table 3. The 5-fold cross-validation results are shown in Table 4, which showed a similar performance as the prediction models conducted. Calibration and DCA curves show that the nomogram has good predictive accuracy and value. The results of calibration are shown in Fig. 4. The DCA curve for predicting 1- and 2-year CSS and OS prediction of SCLC patients with liver metastasis is presented in Fig. 5.

**Table 3:** C-indexes of the prediction models for CSS and OS in SCLC patients with liver metastasis

Models	C-index (95% CI)			
	CSS		OS	
	Training set	Testing set	Training set	Testing set
Total model	0.724 (0.716–0.731)	0.734 (0.722–0.747)	0.732 (0.724–0.739)	0.739 (0.727–0.751)
1-year model	0.727 (0.719–0.734)	0.737 (0.724–0.749)	0.734 (0.727–0.742)	0.742 (0.730–0.754)
2-year model	0.724 (0.717–0.731)	0.735 (0.723–0.747)	0.732 (0.725–0.739)	0.739 (0.727–0.751)

*SCLC* small cell lung cancer, *CSS* cancer specific survival, *OS* overall survival, *CI* confidence interval

**Table 4:** The 5-fold cross-validation results of the performance of the prediction models for CSS and OS in SCLC patients with liver metastasis

5-fold cross-validation	C-index (95% CI)			
	CSS		OS	
	Training set	Validation	Training set	Validation
First fold	0.724 (0.716–0.732)	0.719 (0.703–0.736)	0.732 (0.724–0.739)	0.727 (0.711–0.743)
Second fold	0.725 (0.717–0.733)	0.717 (0.701–0.734)	0.732 (0.724–0.739)	0.730 (0.715–0.746)
Third fold	0.722 (0.714–0.730)	0.728 (0.712–0.744)	0.731 (0.723–0.739)	0.732 (0.717–0.748)
Forth fold	0.723 (0.715–0.731)	0.725 (0.709–0.742)	0.731 (0.723–0.739)	0.732 (0.716–0.748)
Fifth fold	0.723 (0.715–0.732)	0.722 (0.706–0.738)	0.735 (0.727–0.743)	0.729 (0.713–0.745)

*SCLC* small cell lung cancer, *CSS* cancer specific survival, *OS* overall survival, *CI* confidence interval

### Comparisons of prediction models with and without chemotherapy for predicting CSS and OS in SCLC patients with liver metastasis

The nomogram indicated that chemotherapy carries significantly more importance compared to other variables, thereby, comparisons of prediction models with and without chemotherapy for predicting CSS and OS in SCLC patients with liver metastasis were performed. The C-index of the prediction models with only chemotherapy was 0.687 (95% CI: 0.681–0.693) for predicting CSS in the training set, and the C-index of the prediction models without chemotherapy was 0.613 (95% CI: 0.604–0.621) (Tables 5 and 6).

**Table 5:** Performance of the prediction models with and without chemotherapy for predicting CSS and OS in SCLC patients with liver metastasis

Models	C-index (95% CI)			
	CSS		OS	
	Training set	Testing set	Training set	Testing set
Total model	0.724 (0.716–0.731)	0.734 (0.722–0.747)	0.732 (0.724–0.739)	0.739 (0.727–0.751)
Only chemotherapy	0.687 (0.681–0.693)	0.692 (0.682–0.703)	0.692 (0.686–0.698)	0.692 (0.682–0.703)
Total model without chemotherapy	0.613 (0.604–0.621)	0.624 (0.610–0.639)	0.619 (0.610–0.627)	0.624 (0.610–0.639)

*SCLC* small cell lung cancer, *CSS* cancer specific survival, *OS* overall survival, *CI* confidence interval

**Table 6:** Comparisons of prediction models with and without chemotherapy for predicting CSS and OS in SCLC patients with liver metastasis

Models	CSS	OS
	*P*	*P*
Total model vs Only chemotherapy	< 0.001	< 0.001
Total model vs Total model without chemotherapy	< 0.001	< 0.001

*SCLC* small cell lung cancer, *CSS* cancer specific survival, *OS* overall survival

## Discussion

In this study, prediction models were constructed to predict the CSS and OS in SCLC patients with liver metastases. The results showed that the prediction models exhibited good performance with the C-indexes being 0.725 of CSS in SCLC patients with liver metastases and OS being 0.732. Our result indicated that age, the male, married, N stage, other metastases, chemotherapy, and radiotherapy were independent predictive factors affecting the CSS and OS of SCLC patients with liver metastasis.

A number of existing nomograms have been conducted for patients with SCLC. However, most of these models, have been developed for different stages of diagnosed SCLC, and none have included liver metastasis. In a single institution study by Xiao et al., a nomogram was constructed to predict the 3-year and 5-year OS for SCLC [18]. However, the c-index of the nomogram (= 0.60) was not high, and data on T, N, and M information were missing. Xie et al. [19] established two nomograms by classifying patients as limited or extensive SCLC, without including tumor pathological information such as tumor size. Pan et al. [20] established a nomogram for SCLC, using only a small sample size of resected SCLC patients. Gao et al. establish a prediction model for extensive-stage SCLC patients with different metastatic sites [12]. However, the C-index was 0.66. We first used the SEER database to identify independent factors for CSS and OS, and establish the prediction models for the survival of SCLC with liver metastasis. The C index of nomograms is greater than 0.7, indicating that it has sufficient discriminatory power. The DCA results showed that the nomogram we established had good clinical utility. The nomogram prediction model of SCLC liver metastasis may help to clarify the treatment stratification and efficacy evaluation of SCLC liver metastasis. Using our prediction models, researchers and clinicians could easily predict the CSS and OS of each SCLC patient with liver metastasis.

Previous studies have shown that advanced age is a poor prognostic factor for SCLC patients [9, 13, 21]. Our study suggests that elderly SCLC patients diagnosed with liver metastases have unfavorable CSS and OS. The increased risk may be associated with degenerative changes in various aspects of organ function and with an increased incidence of comorbidities [22]. In addition, older patients may be more susceptible to toxic reactions caused by systemic treatment, while younger patients may be in better health and better able to tolerate the side effects of chemotherapy and radiotherapy [23]. In this study, being unmarried and male are poor prognostic factors for SCLC patients with liver metastasis. Unmarried patients lack the psychological and economic support of their spouses, which leads to poor prognosis [24]. In terms of treatment methods, radiotherapy and chemotherapy are common treatment methods for SCLC [25]. Chemotherapy, as the main treatment for SCLC, has been proven to prolong survival time [26]. Gao et al. [12] also reported that chemotherapy was a predictive variable of prognosis for extensive-stage SCLC patients. Radiotherapy is usually considered as a palliative local treatment, mainly used for symptomatic treatment [27]. In this study, radiotherapy was significantly associated with prolonged CSS and OS of SCLC patients with liver metastases. Selecting a more precise treatment for patients, avoiding wasting healthcare resources, and guiding clinicians in their treatment decisions is of importance.

At present, research into SCLC patients with liver metastases is limited or only has focused on a special type of lung cancer patient. Combined with the fact that liver metastasis of SCLC patients is relatively high and liver metastasis has a negative impact on prognosis, research on liver metastasis of SCLC patients is very necessary and urgent. To the best of our knowledge, this is the first study that constructed nomograms to predict the CSS and OS of SCLC patients with liver metastasis. In this paper, based on the SEER database, a large number of related information of SCLC patients with liver metastasis was extracted to make the study more widely applicable. Second, oncologists and patients alike want reliable prognostic information for each patient. One of the tools that can achieve this is the nomogram, which creates a simple graphical representation of a statistical prediction model to generate numerical probabilities of clinical events. We established not only the OS prediction model of liver metastasis in SCLC patients, but also the CSS model of liver metastasis in SCLC patients, which can be used by clinicians to predict the OS and CSS of liver metastasis in SCLC patients respectively, thus, improving the survival of SCLC patients with liver metastasis.

The limitations of this study should be acknowledged. First, this study is a retrospective study and has its own limitations, which may have influenced our results. Second, due to the lack of information on living environment, lifestyle, adjuvant therapy and commodities, it is impossible to consider all prognostic factors comprehensively, which is also an inherent limitation of the SEER research. Third, no external validation was performed to further evaluate this nomogram, possibly limiting the generalization of our model. Future well-designed prospective studies with large sample sizes are needed to validate the results of this study.

## Conclusion

In this study, the prediction models showed excellent predictive performance for predicting survival of SCLC patients with liver metastasis. Clinicians can predict CSS and OS of SCLC patients with liver metastasis by simply incorporating prognostic factors into a nomogram. Early identification of high-risk groups with poor prognoses can enable personalized intervention, improve patient survival.

### Supplementary Information

**Additional file 1.**

## Data Availability

All data relevant to the study are included in the article is available from SEER database, https://seer.cancer.gov/.

## Abbreviations

SCLC: Small cell lung cancer
OS: Overall survival
CSS: Cancer-specific survival
SEER: Surveillance, Epidemiology, and End Results
ICD-O-3: International Classification of Diseases for Oncology third edition
NOS: Not otherwise specified
Mean ± SD: Mean ± standard deviation
ANOVA: Analysis of one-way variance
HR: Hazard ratio
CI: Confidence interval
